# Development and implementation of an etiology-based diagnostic framework for acute abdominal pain in emergency settings

**DOI:** 10.1186/s41065-025-00507-3

**Published:** 2025-07-18

**Authors:** Hui Guo, Xu-Rui Li, Yun-Lei Du, Yang-Juan Jia, Hong-Ling Li, Qian Zhao, Yan-Peng Li, Jian-Guo Li

**Affiliations:** Department of Emergency, Hebei General Hospital, No.348 West Heping Boulevard, Shijiazhuang, Hebei Province 050051 China

**Keywords:** Abdominal pain, Acute renal infarction, Checklist, Clinical decision, Cognitive load, Cognitive task analysis

## Abstract

**Background:**

Diagnostic checklists have been demonstrated to reduce errors in clinical reasoning. Building on previous validation studies, this research presents the development and clinical application of an etiology-based diagnostic framework for evaluating acute abdominal pain. The framework integrates a structured checklist of abdominal pain etiologies with a process-oriented diagnostic strategy, aiming to enhance diagnostic accuracy and clinical outcomes. This approach also serves as a potential model for the creation of diagnostic tools applicable to other symptom complexes encountered in emergency medicine.

**Methods:**

A cognitive task analysis (CTA) was conducted with participation from five emergency medicine experts employing a think-aloud methodology. The experts described their diagnostic reasoning processes and queried relevant clinical data to extract foundational diagnostic principles. Based on these findings, a checklist categorizing etiologies of abdominal pain was constructed, drawing from anatomical and diagnostic considerations. The clinical utility of the checklist was evaluated through its application to a representative complex case.

**Results:**

The diagnostic checklist was organized into five principal etiological categories: local organ disorders, diseases of adjacent organs, systemic diseases, psychogenic disorders, and gynecological conditions. Its implementation facilitated the accurate identification of atypical acute renal infarction in a diagnostically challenging case, enabling prompt clinical intervention.

**Conclusions:**

CTA provides a robust method for modeling expert diagnostic reasoning and supports the development of structured, etiology-based diagnostic tools. This framework enhances diagnostic precision for individuals presenting with acute abdominal pain in emergency settings and may inform the development of similar tools for other clinical presentations.

## Introduction

Acute abdominal pain is among the most frequently encountered symptoms in emergency medicine. In the United States and Europe, approximately 8–9% of patients presenting to emergency departments report abdominal pain as a chief complaint [1]. The etiology of acute abdominal pain is often complex, with potential causes arising from both intra-abdominal and extra-abdominal sources, involving multiple organ systems. The majority of cases are characterized by a sudden onset and rapid progression, and may involve various life-threatening conditions [2]. Consequently, the diagnostic process is frequently complicated by uncertainty and the risk of diagnostic error [3, 4]. 

Although the *Practice Guidelines for Primary Care of Acute Abdomen* (2015) developed by the Japanese Society for Abdominal Emergency Medicine and the Dutch *Guideline for the Diagnostic Pathway in Patients with Acute Abdominal Pain* provide structured approaches to diagnosis [5, 6], their practical applicability in emergency settings remains limited. This limitation is primarily attributed to the inherent complexity of abdominal pain etiologies and the high cognitive demands of emergency environments, which may contribute to delays, misdiagnoses, or missed diagnoses [7]. As a result, these guidelines are not consistently applied in routine clinical practice, and no universally adopted, clinically feasible diagnostic framework currently exists for abdominal pain, making effective diagnosis a persistent challenge.

Cognitive task analysis (CTA) was introduced into the healthcare field in the 1960s in the United States as a methodology to systematically extract expert knowledge by deconstructing complex tasks into essential steps and identifying the corresponding cognitive processes. CTA also uncovers decision-making elements that may not be directly observable [8]. Previous research has shown that expert clinicians possess distinct competencies in information gathering and clinical decision-making [9]. Through repeated clinical exposure, these individuals develop structured diagnostic schemas that enable efficient processing of core diagnostic elements and execution of high-level differential diagnoses [10]. 

Based on these findings, it was hypothesized that applying CTA could aid in refining expert diagnostic strategies and knowledge structures, potentially allowing both novice and experienced clinicians to achieve improved diagnostic performance in cases of acute abdominal pain. This hypothesis was preliminarily supported by previous research demonstrating that a novel diagnostic model incorporating an etiology-based checklist and process-oriented reasoning was associated with enhanced diagnostic accuracy and improved patient outcomes [4]. Additional studies have further established the utility of diagnostic checklists in reducing errors [11]. 

The present study aims to describe the methodology used in developing an etiology-based checklist for causes of abdominal pain, illustrate its clinical application through a case of atypical acute renal infarction, and examine the cognitive mechanisms underlying its effectiveness. The objective is to contribute to the development of diagnostic frameworks for other emergency department presentations and support the standardization of diagnostic processes in acute care settings.

## Methods

### Ethical considerations

This study received approval from the institutional ethics committee. Written informed consent was obtained from all participants or, where applicable, from their legal guardians.

### Development of an etiology-based checklist for abdominal pain

**Fig. 1:**
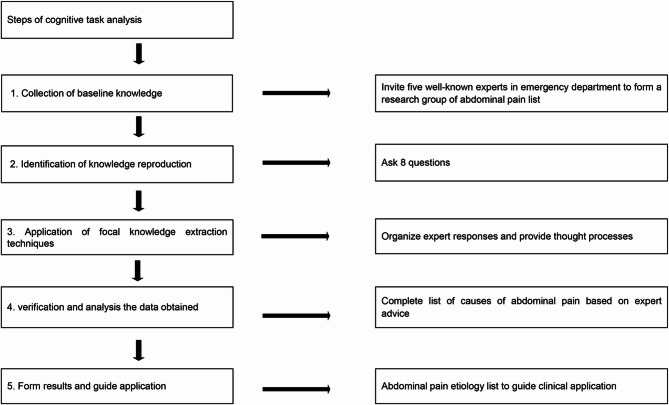
Flowchart illustrating the development of an etiology-based checklist for abdominal pain using CTA

The etiology-based checklist for abdominal pain was developed using CTA methodologies [8, 11]. The CTA framework comprised four key components: (1) observations and interviews, wherein participants were observed and interviewed while performing diagnostic tasks; (2) process tracing, involving the articulation of thought processes either during or immediately following task execution; (3) conceptual techniques, which facilitated a detailed representation of the conceptual structure and interrelationships within the diagnostic domain; and (4) formal modeling, in which modeled tasks were introduced into cognitive frameworks and evaluated for outcome efficacy (Fig. 1).

The study population included adults presenting to the emergency department with common presentations of acute abdominal pain. Inclusion criteria comprised symptom duration of less than one week and a high likelihood of requiring urgent medical or surgical intervention [6]. Common accompanying symptoms included nausea, vomiting, hematemesis, hematochezia, diarrhea, arrhythmias, and hypotension.

Five senior emergency medicine experts were invited to participate in the study, including three deputy chief physicians and two chief physicians. All participants held master’s degrees and had extensive clinical experience. The group consisted of four males and one female, with a mean age of 51.4 ± 5.28 years and a mean clinical experience of 27.2 ± 5.36 years.

Three primary objectives guided the development of the checklist: (1) integration of foundational biomedical sciences with clinical practice, grounded in the pathophysiological mechanisms of abdominal pain; (2) development of a diagnostic model that adheres to principles of anatomical localization; and (3) formulation of a clinical pathway consistent with emergency medicine protocols. To address these goals and reflect the realities of emergency clinical workflows, eight structured questions were formulated (Table 1).

**Table 1:** Key issues of the expert’s program

(1) Patient had life-threatening diseases/conditions?
(2) What is the definition of abdominal pain as a clinical presentation?
(3) Is abdominal pain the main problem (symptom) of this patient?
(4) Is this patient at high-risk symptoms?
(5) What are the possible causes of abdominal pain (direction)?
(6) What is the most likely diagnosis for this patient? (specific disease)
(7) Does this patient have any other problems?
(8) Does this patient’s diagnosis explain all clinical manifestations?

Each expert independently responded to the structured questions, referring to standard textbooks and authoritative clinical literature, with emphasis on internal medicine, diagnostic methodology, anatomy, and relevant guidelines. The responses were subsequently reviewed in a consensus meeting to synthesize expert reasoning strategies. Based on this discussion, a comprehensive checklist of differential diagnoses for abdominal pain was constructed.

To further refine the checklist, a trained researcher conducted structured “diagnostic clue recall” interviews with each expert. This process involved eliciting detailed recall of diagnostic strategies related to abdominal pain and was used to organize and consolidate the collective diagnostic reasoning. Preliminary categorization of disease etiologies was informed by pathophysiological principles and further refined using anatomical localization criteria. Differential diagnoses were prioritized based on prevalence and clinical importance. A de-escalation reasoning approach was adopted, and critical illnesses were identified using “red flag” indicators consistent with emergency diagnostic standards [12]. 

### Design of the diagnostic and treatment model for acute abdominal pain

CTA was utilized to construct a diagnostic framework for acute abdominal pain. The design incorporated triage principles outlined in the *Guidelines for Grading Emergency Patients* issued by the Ministry of Health in August 2011. Differential diagnosis was guided by a de-escalation reasoning strategy applied to a reclassified etiology checklist [13]. 

The checklist was developed in accordance with three core principles: simplicity, clinical effectiveness, and operational measurability. Corresponding diagnostic procedures were aligned with each checklist category to support structured evaluation and clinical decision-making [14]. 

Diagnosis and treatment pathways were stratified based on patient acuity, enabling prompt identification and evaluation of potentially life-threatening conditions listed in the etiology checklist. For patients assessed to be in critical condition, immediate diagnostic and therapeutic interventions were initiated as part of the initial clinical management.

## Results

### Etiology-based checklist for abdominal pain

The developed checklist classified the causes of abdominal pain into five primary etiological categories:

(1) Local Organ Disorders– This category included conditions affecting the abdominal wall, such as inflammatory, neuropathic, and structural disorders (e.g., abdominal wall contusion, abscess, and herpes zoster). Disorders of the gastrointestinal tract were also categorized here under “celiac disease,” encompassing functional gastrointestinal disorders (e.g., gastrointestinal spasm, irritable bowel syndrome), acute inflammation, hollow organ perforation, luminal obstruction, organ torsion, and intra-abdominal vascular pathologies. This category represented a central focus in the differential diagnosis process (Table 2).

**Table 2:** Checklist of causes of abdominal pain

Concept	Anatomical positioning		Etiological classification	Acute common disease
Onset < one week	Local organ	Abdominal cavity	1. Acute inflammation	Acute gastroenteritis, hemorrhagic necrotizing enteritis, pancreatitis, cholecystitis, suppurative obstructive cholecystitis Ο, reflux esophagitis, spontaneous peritonitis, urinary tract infection, etc.
			2. Organ rupture	Liver rupture Ο, spleen rupture Ο, duodenal ulcer perforation Ο, etc.
			3. Obstructed or twisted organs	Intestinal obstruction, bile duct stones, biliary ascariasis, renal ureteral stone obstruction, volvulus Ο, mesenteric or omentum volvulus Ο, etc.
			4. Intraperitoneal vascular disease	Mesenteric artery embolization Ο, portal vein embolism Ο, spleen embolism Ο, renal embolism Ο, abdominal aortic dissection Ο, etc.
			5. Functional diseases	Gastrointestinal cramps, irritable bowel syndrome
		Abdominal wall	6. Inflammation or Neuropathy	Abdominal wall contusion, abscess, abdominal wall herpes zoster, other abdominal wall diseases, etc.
	Adjacent organs		7.Cardiopulmonary spine	Angina pectoris, myocardial infarction Ο, acute pericarditis, pneumonia, pulmonary infarction Ο, pleurisy, hiatal hernia, thoracic spine tuberculosis or tumor, etc.
	Systemic		8. Systemic disease	Such as ketoacidosis Ο, abdominal allergic purpura, abdominal epilepsy, uremia, hematoporphyria, systemic lupus erythematosus, etc.
	Psychogenic		9. Psychogenic disease	Abdominal neurosis
	Gynecology		10. Gynecological and Fertility Related Disorders	Ectopic pregnancy Ο, ovarian cyst torsion Ο, ovarian rupture Ο, pelvic inflammatory disease, ovarian hemorrhage, etc.

(2) Diseases of Adjacent Organs– This category included pathologies of organs adjacent to the abdominal cavity, such as the heart, mediastinum, lungs, and spine. Representative conditions included angina pectoris, acute myocardial infarction, pericarditis, pneumonia, pulmonary infarction, pleurisy, hiatal hernia, and thoracic spinal tuberculosis or neoplasms.

(3) Systemic Diseases– This group encompassed hematologic disorders, immune-mediated diseases, toxic exposures, endocrine dysfunctions, and metabolic disturbances. Examples included diabetic ketoacidosis, abdominal manifestations of Henoch–Schönlein purpura, abdominal epilepsy, uremia, porphyria, and systemic lupus erythematosus.

(4) Psychogenic Disorders– Conditions categorized under this heading included abdominal neurosis and other somatoform disorders.

(5) Gynecological Conditions– Recognized as a distinct etiological group to aid recall and differentiation, this category included ectopic pregnancy, ovarian torsion, ovarian cyst rupture, pelvic inflammatory disease, and ovarian hemorrhage.

### Diagnostic and treatment model for acute abdominal pain

**Fig. 2:**
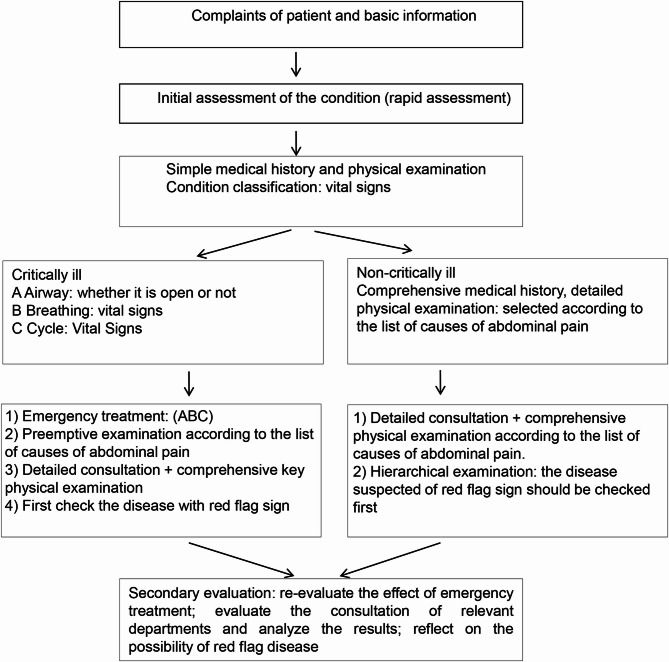
Emergency diagnostic and treatment workflow for patients presenting with acute abdominal pain, structured according to clinical urgency and guided by the etiology-based checklist

For patients presenting with potentially life-threatening abdominal pain, the diagnostic process was prioritized according to clinical urgency, likelihood of specific etiologies, and feasibility of intervention (Fig. 2). In non-critical cases, the checklist functioned as a structured decision-support tool. Clinical assessment began with systematic history taking and physical examination, followed by stratified screening and the selective application of laboratory tests and diagnostic imaging as indicated [4–6]. 

### Case presentation

A 77-year-old female presented with a two-day history of persistent dull pain in the left lower quadrant of the abdomen. The pain was not influenced by movement or body position and was associated with nausea but no vomiting. Initial evaluation at a local facility revealed atrial fibrillation, leukocytosis, and a mildly elevated D-dimer level. Due to the lack of symptom relief with analgesics and antispasmodics, the patient was referred for further evaluation.

On presentation, vital signs were as follows: temperature 37.2 °C, pulse rate 123 beats/min, respiratory rate 16 breaths/min, blood pressure 114/77 mmHg, and oxygen saturation 97% on room air. The Visual Analogue Scale (VAS) pain score was 6. The patient was conscious but demonstrated reduced responsiveness. No cutaneous abnormalities were observed. Pupils were equal, round, and reactive to light. According to the emergency triage classification system [12], the condition was assessed as non-life-threatening.

A hierarchical approach to clinical history taking and physical examination was implemented based on the etiology-based checklist. The structured diagnostic pathway considered five domains: local organ disorders, diseases of adjacent organs, systemic diseases, psychogenic disorders, and gynecological conditions.

Psychogenic disorders were excluded due to the patient’s intact consciousness and absence of psychiatric history. Gynecological causes were ruled out based on a postmenopausal status of 20 years. The absence of herpetiform lesions and a normal abdominal wall examination excluded cutaneous or abdominal wall disorders. Given the ineffective response to antispasmodics, functional gastrointestinal conditions were also excluded.

Pain localization to the left lower quadrant prompted consideration of disorders involving the descending and sigmoid colon and the left kidney. However, the physical examination revealed no abdominal tenderness, rebound, or guarding. Liver and spleen were non-palpable, bowel sounds were normal, and bilateral renal percussion tenderness was absent. McBurney’s point and Murphy’s sign were negative, making gastrointestinal rupture, torsion, or obstruction less likely.

Blood analysis, urinalysis, and serum amylase testing were performed to evaluate for intra-abdominal infection. Abdominal computed tomography (CT) was considered necessary to confirm or exclude structural abnormalities. The patient’s known history of atrial fibrillation with a rapid ventricular response presented a potential dissociation between clinical symptoms and physical findings, which did not exclude the possibility of intra-abdominal vascular pathology. Although the abdominal pain had persisted for two days without classic signs of intestinal involvement, it was recognized that such symptoms may present atypically in older adults. Consequently, thromboembolic vascular disease was suspected, and D-dimer testing was performed.

The patient reported no chest pain, cough, or sputum production. Bilateral breath sounds were clear on auscultation. Despite the presence of arrhythmia, no cardiac murmurs were detected. Given the patient’s age, an electrocardiogram (ECG) was conducted to evaluate for potential cardiopulmonary causes of abdominal pain. The spinal percussion test was negative, allowing for temporary exclusion of spinal and adjacent structural etiologies. The absence of significant comorbidities or medication use reduced the likelihood of systemic contributors; nonetheless, basic laboratory testing was performed to exclude systemic disease. These included assessments of hepatic and renal function and serum glucose levels.

Test results obtained within 30 to 60 min were as follows: white blood cell count 14.67 × 10⁹/L (reference range: 3.5–9.5 × 10⁹/L), neutrophil percentage 85.60% (40–75%), hemoglobin 130 g/L (115–150 g/L), and platelet count 198 × 10⁹/L (125–350 × 10⁹/L). Serum amylase was within normal range at 72 U/L (35–135 U/L). ECG confirmed atrial fibrillation with a rapid ventricular response. Liver and kidney function tests and urinalysis yielded normal results. Coagulation testing revealed an elevated D-dimer level of 1.12 mg/L FEU (0–0.55 mg/L FEU), with a blood glucose level of 5.94 mmol/L (3.9–6.1 mmol/L). Additional laboratory findings included potassium 4.1 mmol/L (3.5–5.3 mmol/L), sodium 127 mmol/L (137–147 mmol/L), chloride 97 mmol/L (99–110 mmol/L), and creatinine 93 µmol/L (41–81 µmol/L). Coagulation parameters included a prothrombin time of 12.1 s (9.8–12.1 s), an international normalized ratio of 1.05 (0.85–1.3), and a fibrinogen level of 3.2 g/L (2–4 g/L). These results did not support systemic disease as the underlying etiology of the abdominal pain.

**Fig. 3:**
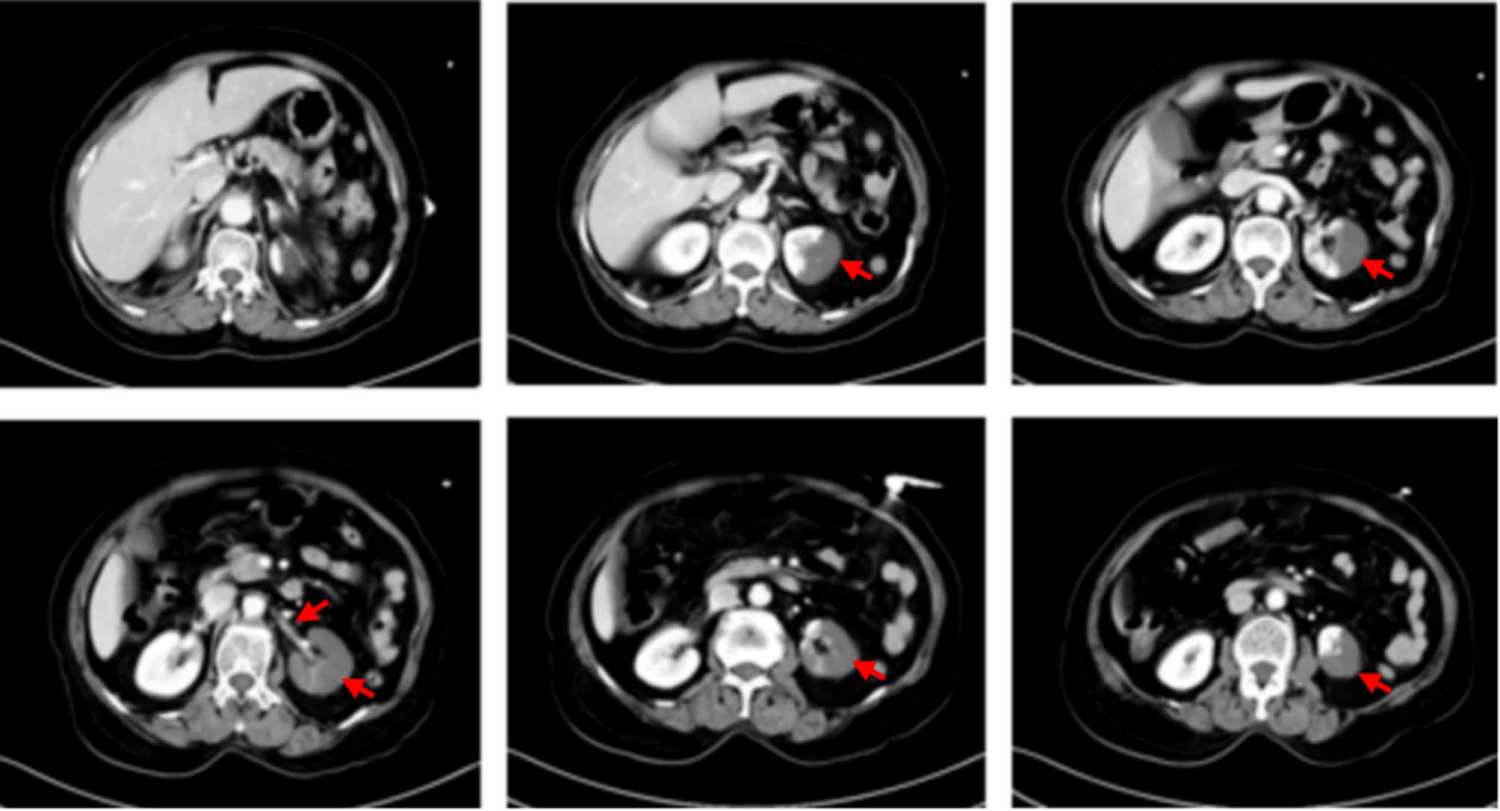
Contrast-enhanced CT of the abdomen and pelvis. The main trunk of the left renal artery appears narrower than the right, and a large region of the left kidney shows no contrast enhancement, indicating infarction (highlighted by the arrow)

Based on the checklist and clinical analysis, inflammatory and vascular conditions remained in the differential diagnosis. Given the potential severity of intra-abdominal vascular lesions, designated as “red flag” conditions, an abdominal and pelvic CT scan with contrast was performed to further evaluate for inflammatory pathology or structural disruption, including organ torsion or rupture. Imaging revealed narrowing of the main trunk of the left renal artery and thrombosis in a distal branch, resulting in significant infarction of the left kidney. The mesenteric vasculature appeared patent with no evidence of embolism. These findings supported a diagnosis of renal artery embolism. The complete diagnostic process was completed within 90 min (Fig. 3), and the patient was subsequently admitted to the vascular surgery department for further management.

## Discussion

Diagnosis is a multifaceted cognitive process involving multiple stages, making it inherently complex. In emergency medicine, the predominant reasoning model is hypothetical-deductive reasoning, which operates under a model-comparison framework [15]. This approach, including for abdominal pain, often relies on the prior experience of clinicians to facilitate rapid identification of common conditions. However, it is vulnerable to more than 100 recognized cognitive biases associated with heuristic thinking [16]. To reduce diagnostic errors attributable to cognitive bias, some scholars have proposed incorporating systematic “slow thinking” and structured hypothesis-deductive reasoning frameworks into clinical practice [17]. While such approaches are logic-driven and grounded in an understanding of disease mechanisms, they are cognitively demanding and time-intensive, challenges that often conflict with the operational constraints of emergency care.

Previous research has indicated that expert clinicians employ diagnostic “shortcuts” or heuristics to improve efficiency [18]. Schmidt and Boshuizen have described how experienced physicians consolidate clinical knowledge into organized “illness scripts,” allowing for more effective information retrieval and decision-making [19]. By articulating these decision trees and heuristic frameworks, novice clinicians can be trained to emulate expert-level reasoning, thereby reducing diagnostic errors [10]. 

In this study, diagnostic clue recall interviews were conducted using CTA to capture expert reasoning and refine cognitive strategies specific to abdominal pain diagnosis [20]. 

Traditionally, the abdomen is divided into nine anatomical regions to aid in diagnosis [21]. However, the etiological spectrum of abdominal pain extends beyond anatomical boundaries and includes local, systemic, and referred conditions. Textbook classifications of acute abdominal pain are often highly detailed and fragmented, making it challenging for clinicians to integrate this knowledge into practical diagnostic workflows. Based on expert cognitive models, the etiological framework of acute abdominal pain was restructured into five broad categories: local organ disorders, diseases of adjacent organs, systemic diseases, psychogenic disorders, and gynecological conditions. This reclassification was intended to support comprehensive, structured reasoning and enhance the efficiency of differential diagnosis in emergency settings [4]. 

Celiac vascular pathologies, such as acute renal infarction, pose significant diagnostic challenges. Acute renal infarction is rare, accounting for approximately 0.004% of emergency department visits. Although flank pain occurs in approximately 71.2% of cases, the clinical presentation is typically nonspecific and may lack confirmatory laboratory findings [22]. Hematuria, while suggestive, is inconsistently observed, and some patients remain asymptomatic [23]. In the present case, the patient experienced persistent left lower quadrant abdominal pain without hematuria, contributing to the diagnostic difficulty. Conventional model comparison approaches, when applied without structured support, may lead to delayed recognition of atypical or rare conditions. However, the expert-informed framework emphasized structured reasoning via the checklist, guiding clinicians to systematically collect history, symptoms, and preliminary test results within five etiological categories. Horizontal comparison across categories facilitated systematic exclusion of more common conditions and highlighted “vascular abdominal pain” as a high-risk diagnosis associated with a red flag indicator. As a result, angiographic imaging was prioritized over non-contrast abdominal CT, expediting diagnosis and reducing diagnostic delay. The diagnostic process was completed within 90 min.

This structured approach reduces cognitive load, which is particularly significant in emergency medicine, where physicians face high information-processing demands and time constraints [14]. The cognitive demands faced by medical professionals, particularly emergency physicians, are demonstrably higher than those required in many other complex professional tasks [24]. Cognitive psychology research suggests that diagnostic accuracy and speed rely on clinicians’ accumulated “disease scripts”—structured mental models built through repeated clinical exposure [25]. Expert clinicians typically possess a broader repertoire of such scripts, which can be efficiently accessed during diagnostic reasoning [26]. In contrast, early-career physicians often rely on limited short-term memory, increasing their susceptibility to diagnostic errors due to cognitive overload [24]. 

The checklist developed in this study supports clinical reasoning by organizing differential diagnoses into five key etiological categories. This structure aids in rapid pattern recognition and systematic exclusion of conditions, thereby improving diagnostic accuracy and efficiency. The value of diagnostic checklists has been well documented, notably by Gawande [14], and validated by multiple studies showing their utility in improving efficiency, particularly in critical care environments [27, 28]. Ely et al. have similarly reported the benefits of checklist use in general practice, demonstrating reductions in diagnostic error and enhanced decision-making consistency [29]. 

The checklist is not intended as a rigid protocol but rather as a cognitive aid designed to prompt structured evaluation of potential causes based on real-time clinical reasoning. If initial screening yields no clear diagnosis, clinicians are encouraged to revisit each of the five diagnostic categories using the accumulated clinical data. This iterative process promotes structured reflection, a practice shown to improve diagnostic performance [21, 24]. This model aligns with diagnostic and treatment strategies adopted by institutions such as the University of Calgary School of Medicine [30]. 

Several limitations should be acknowledged. First, the categorization of the checklist is relatively general and may not comprehensively capture all causes of abdominal pain. Second, while the tool offers structured support, clinical judgment remains central to its effective application. Third, current validation is based on a single-center retrospective study. There is a need for prospective, multi-center studies, including evaluations of health economic impact, to establish the broader applicability and long-term effectiveness of this diagnostic framework.

## Conclusions

In summary, the etiology-based checklist for abdominal pain developed in this study provides a structured, clinically practical, and cognitively accessible tool to support diagnostic reasoning. Its design, grounded in CTA, captures expert diagnostic strategies, enabling the systematic organization of abdominal pain etiologies and corresponding diagnostic processes. This framework enhances diagnostic accuracy and efficiency in the evaluation of acute abdominal pain and may serve as a reference model for developing similar diagnostic tools for other emergency presentations.

## Data Availability

All data generated or analyzed during this study are included in this article.

## Abbreviations

CTA: Cognitive task analysis
ARI: Acute renal infarction
CT: Computed tomography
